# Patient and clinician experiences of language barriers in Qatar's multilingual public mental health care: a qualitative study

**DOI:** 10.1080/17482631.2026.2722431

**Published:** 2026-08-24

**Authors:** Dalia Alkhatib, James Currie, Shuja Reagu, Majid Alabdulla

**Affiliations:** a The Mental Health Service, Hamad Medical Corporation, Doha, Qatar; b College of Humanities and Social Sciences, Hamad Bin Khalifa University, Doha, Qatar; c Cardiff University, Cardiff, United Kingdom; d Birmingham and Solihull Mental Health NHS Foundation Trust, Birmingham, United Kingdom; e College of Medicine, Qatar University, Doha, Qatar

**Keywords:** Communication, interpreter, language barriers, mental health, therapeutic relationship

## Abstract

**Purpose:**

Effective communication is central to mental health care, yet language barriers in multilingual settings can compromise assessment, treatment, and therapeutic relationships. Qatar’s Mental Health Service (MHS) at Hamad Medical Corporation serves a largely Arab patient population, while many clinicians use English as their primary working language. This study aims to examine how language barriers affect care delivery and the experiences of patients and clinicians within MHS.

**Method:**

This qualitative study adopted facet methodology, generating data through semi-structured interviews with psychiatrists, psychologists, and patients, onsite non-participant observations, and institutional archives. Data were analyzed using practical thematic analysis guided by interpretive description.

**Results:**

Findings indicate limited systematic language assistance and reliance on ad hoc interpreters, a practice that diverges from institutional policy. Clinicians described how inadequate interpretation compromised assessment accuracy, patient safety, and therapeutic relationship, and generated emotional strain and reduced professional efficacy. Patients described difficulty articulating their concerns in English and viewed language-concordant care as central to protecting privacy and emotional safety. Participants across both groups called for professional interpreters, language-concordant care, staff language training, and AI-assisted interpreting tools as potential improvements.

**Conclusions:**

Language barriers represent a central challenge in public mental health care in Qatar, particularly where ad hoc interpreting becomes routine. More structured, context-sensitive language support is needed in multilingual mental health settings.

## Introduction

Effective communication in healthcare lies at the heart of achieving therapeutic efficacy and quality care (Arkorful et al., 2021; Sharkiya, 2023). In multilingual contexts, however, communication is compromised when healthcare providers and patients do not share proficiency in the same language and appropriate language assistance is unavailable (Meuter et al., 2015). Available research demonstrates that language barriers are associated with lower patient engagement with recommended healthcare (Morrison et al., 2012), increased risk of inaccurate assessment and adverse events (Divi et al., 2007), higher risk of medical errors with serious consequences (Jacobs et al., 2006) and reduced access to healthcare services (Rajiv et al., 2021). Such impact can be further exacerbated in mental health settings, where diagnostic and therapeutic processes rely heavily on verbal interaction and nuanced communication between clinicians and their patients (Kilian et al., 2015; Ohtani et al., 2015; Richly, 2025).

In response to language barriers, some healthcare systems employ professional interpreters to facilitate communication (Al Shamsi et al., 2020; Hieke et al., 2024; Karliner et al., 2007; Rosenberg et al., 2007), while others rely on ad hoc interpreters, including family members or bilingual staff, to bridge linguistic gaps (Hannouna, 2012; Osae-Larbi, 2016; Rosenberg et al., 2007). Within the Gulf Cooperation Council (GCC) region, the presence of large expatriate populations has generated multilingual and multicultural service environments, including healthcare (Erumban & Al-Mejren, 2024), creating a context in which language barriers in clinical care have attracted growing regional research attention. Saudi–focused studies indicate that language barriers adversely affect communication, patient safety, and satisfaction (Al Shamsi et al., 2020; Al-Khathami et al., 2010; Alanazi, 2023; Alhamami, 2022), with recommendations including the use of professional interpreters, electronic translation systems, online translation tools, and targeted language training for healthcare workers (Al Shamsi et al., 2020; Al-Khathami et al., 2010; Alanazi, 2023; Alhamami, 2022). Evidence from Bahrain similarly identifies language discordance as a barrier to nurse–patient communication and the delivery of quality care (Abdulla et al., 2022; Vaithinathan et al., 2022). In Kuwait, exploratory research suggests that language barriers contribute to negative patient experiences, misdiagnosis, and delays in care (Nuguid & Muir, 2019). In the United Arab Emirates, studies have highlighted the emotional and professional strain experienced by non-Arab healthcare providers due to language barriers, alongside calls for adequate interpreting services (Al-Yateem et al., 2023; Hannouna, 2012).

Within the Qatari context, the rapid expansion of the healthcare system in alignment with Qatar National Vision 2030 has introduced similar challenges, particularly due to the country’s reliance on internationally trained healthcare professionals and a limited national workforce (Al-Kuwari et al., 2021; Tan et al., 2014). As the primary public healthcare provider, Hamad Medical Corporation (HMC) serves Qatar’s population, including both Qatari nationals and a predominantly expatriate population (Rahman et al., 2023).

Scholars investigating language discordance within HMC suggest that language gaps within this context are often addressed through ad hoc interpreters or bystanders (Abdelrahim et al., 2017; Kamal, 2015; Kanoun et al., 2024), with healthcare providers reporting difficulties in obtaining accurate clinical information and patients expressing frustration with the predominance of English in an Arabic-speaking country (Abdelrahim et al., 2017; Kanoun et al., 2024). Despite these findings, limited attention has been given to the impact of language barriers within Qatar’s Mental Health Service (MHS), the primary public provider of mental healthcare under HMC. This gap is particularly concerning given evidence linking language limitations to patient satisfaction in mental health care (Ghuloum et al., 2010) and the identification of linguistic diversity as a challenge to implementing Qatar’s National Mental Health Strategy (Clelland & Tulley, 2020). In keeping with the goal of delivering world-class care, MHS employs an international mental health workforce in which English is the primary language of communication, and many clinicians do not speak Arabic or have limited Arabic proficiency (LAP). This creates a clear communication challenge, as Arabs constitute the largest patient group treated at the Service (around 69% in 2023), many of whom do not speak English or have limited English proficiency (LEP). Table I shows the nationality distribution of patients treated at the Mental Health Service in 2023.

**Table I.** Nationality distribution of patients treated at the mental health service (2023).

Arab nationalities	%	Non-Arab nationalities	%
Qatar	33.36%	India	9.26%
Egypt	10.60%	Pakistan	6.08%
Sudan	4.52%	Philippines	3.25%
Jordan	4.12%	Bangladesh	2.15%
Syria	3.26%	Iran	1.49%
Palestine	2.39%	Nepal	1.23%
Yemen	2.14%	United Kingdom	1.13%
Lebanon	1.87%	United States	0.90%
Tunisia	1.81%	Sri Lanka	0.83%
Morocco	1.02%	Canada	0.66%
Saudi Arabia	0.84%	Kenya	0.53%
Algeria	0.58%	Turkey	0.28%
Iraq	0.45%	South Africa	0.27%
Somalia	0.37%	Afghanistan	0.22%
Oman	0.36%	Eritrea	0.20%
Bahrain	0.34%	Ethiopia	0.17%
UAE	0.18%	Australia	0.15%
Mauritania	0.15%	Nigeria	0.15%
Libya	0.10%	Uganda	0.15%
Kuwait	0.06%	Indonesia	0.13%
Djibouti	0.03%	Ireland	0.12%
		Brazil	0.12%
		Malaysia	0.12%
		Ukraine	0.11%
		Spain	0.11%
		France	0.10%
		Germany	0.09%
		Italy	0.07%
		Portugal	0.07%
		Mexico	0.06%
		Romania	0.06%
		Ghana	0.06%
		New Zealand	0.06%
		Others^a^	1.19%

^a^ Comprises 71 distinct nationalities, each representing less than 0.05% of the total sample. This category also includes patients with unknown or unrecorded nationalities (0.04%).

Our study examines how language barriers shape the delivery of mental health care and the experiences of patients, psychiatrists and psychologists within the Mental Health Service at Hamad Medical Corporation. To our knowledge, this is the first study to investigate this issue in this specific service, providing an initial empirical account of language-related communication challenges in Qatar’s public mental health context.

## Methods

This qualitative study adopts facet methodology (Mason, 2011) as its overarching research orientation. Data were generated through three methodological facets: semi-structured interviews with patients and healthcare providers, onsite non-participant observations, and archival research. The study followed an inductive reasoning approach, drawing on these facets to examine language barriers across participants’ accounts, observed clinical practices, and institutional archival records (Reichertz, 2007). Written informed consent was obtained from all participants prior to interviews and observations, and participation was voluntary.

### In-depth semi-structured interviews

Semi-structured interviews were employed as one facet, as they are well established in qualitative enquiry for enabling in-depth engagement with research questions and exploring explanatory “why” dimensions (Fylan, 2005). The study sample comprised 13 participants: 7 psychiatrists (Dr), 3 psychologists (Psy), and 3 patients (Pt). The 13 interviews were considered sufficient for the aims and scope of this study, as recurring patterns were evident and no substantially new themes emerged during analysis.

Participants were recruited using snowball sampling through professional contacts within the Mental Health Service (MHS). Healthcare providers at MHS assisted in identifying patients who had experienced language barriers. Interviews were conducted between 7 September 2023 and 7 November 2023. Given the study’s focus on the two most commonly used languages within HMC, Arabic (the official language) and English (the lingua franca), the sample included (1) English-speaking psychiatrists and psychologists who use English as the primary language of communication at MHS and who have limited or no Arabic proficiency, and (2) Arabic-speaking patients who use Arabic as their primary language of communication and who have limited or no English proficiency.

Open-ended questions elicited detailed accounts of participants’ experiences, challenges, and perceived solutions related to language barriers. All interviews were audio-recorded and transcribed verbatim. Arabic interviews were translated into English and back-translated into Arabic to ensure accuracy and preserve meaning. The original and back-translated texts were compared to resolve discrepancies and ensure linguistic equivalence. An independent second translator reviewed the transcripts, and translations were finalised through a consensus-based approach.

### Onsite observation

Onsite non-participant observation formed a second facet, complementing the interviews by allowing participants’ accounts to be considered alongside observed practices in situ. Observation is a well-established qualitative method for examining how individuals act within specific contexts, including routine behaviours and interactional patterns (Darlington & Scott, 2020). This study focused exclusively on encounters in inpatient units and outpatient clinics, which were documented through detailed written field notes rather than audio or video recording.

Eleven sessions (Obs) were observed. Five sessions took place in inpatient units (Male 1, Male 2, and the Female Unit), and six sessions were conducted in outpatient departments across MHS, including the Psychiatry Hospital, Hamad General Hospital, Hazm Mebaireek General Hospital, and the Communicable Disease Centre. Observation sessions were conducted between 24 September 2023 and 14 November 2023. The observations were conducted at varying intervals according to the availability of eligible encounters, rather than according to a predetermined schedule. There was partial overlap between the interview and observation samples, with some clinicians and patients participating in both components of the study. In all sessions, the first author (DA) was introduced to patients and clinicians as a researcher observing the encounter; the observations were therefore overt, and her presence was known to all participants involved in the observed encounters.

A structured checklist was used when ad hoc interpreters were involved in order to assess the quality of interpretation, focusing on clarity, accuracy, transparency, cultural competence, time management, and the overall effectiveness of the interpreter’s presence. To maximise recall accuracy, detailed field notes were written immediately after each session. Observational data functioned as a distinct methodological facet that illuminated the interactional and practical realities of language barriers in situ.

### Archive

Archival research was used as a third facet to examine institutional documentation relevant to communication and language barriers. This facet explored the structural and organisational dimensions of the phenomenon, contributing to the study’s contextual awareness and cautious interpretation of findings. Policies addressing communication and language barriers were retrieved through searching HMC intranet (I-tawasol) which combines all different types of policies related to HMC. Statistics related to MHS patients’ nationalities were obtained with formal approval from the Health Information Management Department.

### Ethical considerations

This study was approved by the institutional review board of Hamad Medical Corporation (MRC-01-23-297). Informed consents were obtained for interviews and observations, ensuring voluntary participation. All participants provided written informed consent prior to participation. They were informed that participation was voluntary and that they could discontinue their participation at any time without penalty or loss of benefits. Requests to withdraw data already collected could be made within seven days of the interview or observation, in which case the relevant audio-recordings, transcripts, coded data, or observational notes would be discarded. Participation or withdrawal had no consequences for clinicians’ employment or patients’ treatment. No financial or other compensation was provided. The study was conducted in accordance with the principles of the Declaration of Helsinki.

### Researcher positionality

All interviews and on-site non-participant observations were conducted solely by the first author (DA), a PhD candidate researching interpreter-mediated mental healthcare and a professional translator-interpreter employed within the Mental Health Service (MHS). DA is a native Arabic speaker, professionally proficient in English, and holds an MA in Translation Studies. She has five years of experience as a professional Arabic–English translator-interpreter. She holds certificates of completion for professional training in medical interpreting and specialised training in mental health interpreting.

Some clinician participants knew DA through task-based professional contact within the service; however, she had no managerial or supervisory authority over them, and these relationships were not personal. The patient participants were not previously known to DA. Clinicians facilitated initial introductions to potential participants, after which participation and consent were discussed directly with them.

DA occupied an insider position in relation to the institutional setting but was an outsider to the specific participant roles under study, as she was neither a clinician nor a patient. Although her professional role included translation and interpreting responsibilities, she did not provide interpretation during any encounter included in the study, including the sessions she subsequently observed.

Her familiarity with the service facilitated access, contextual understanding, and rapport, but it also created the possibility of influencing participants' accounts. Clinician participants, for example, may have been reluctant to criticise institutional practices or may have provided socially desirable responses to a known colleague. These risks were addressed by emphasising that participation was voluntary and confidential and would have no employment or clinical consequences, by discussing potential researcher influence within the research team, and by comparing interview accounts with observational data.

Because the other authors were clinicians working within the same service and might have known some participants, transcripts and extracts shared during collaborative coding and analytical discussions were de-identified to reduce the risk of participant recognition and identification-related influence on the analysis.

### Data analysis

The adoption of the facet methodology orientation enabled the study to explore the multidimensional and entwined experiences of language barriers in mental health care (Mason, 2011).^1^ In alignment with this methodology's principles, the multimethod design - combining semi-structured interviews, observations, and archival research - was not used simply to triangulate data or seek a perfect formulaic fit, but rather to construct strategically designed “facets” that cast light on the problem from different methodological and substantive angles (Mason, 2011). To synthesise and make sense of these diverse insights, the study was guided by interpretive description, which was selected because it is specifically suited to applied health research and supports the generation of practice-relevant understandings of complex experiential phenomena. It values the subjective and experiential knowledge of those with direct clinical or lived experience while enabling individual accounts to be interpreted in relation to broader patterns and contexts (Thorne, 2025).

At the micro-analytical level, the data were systematically processed using practical thematic analysis informed by Saunders et al. (2023), which moves through a rigorous three-step process of reading, coding, and collaborative theming. All data collected from the three methodological facets were converted into written text ready for analysis; recorded interviews were transcribed, and observations were documented as detailed written field notes. During the reading phase, the data were reviewed, and summary notes were written to capture initial impressions. In the coding phase, the first author (DA) with guidance from and discussion with the second author (JC) generated initial full-thought codes for the data sets, which were then discussed and refined among all authors to ensure multiple perspectives were considered. The codes were then reviewed through ongoing discussion among the authors and refined into broader themes. Following Saunders et al., practical thematic analysis, these themes were framed as descriptive, complete thoughts that could be expressed as full-sentence analytic claims, rather than as broad categories or topic labels. A coding table was maintained throughout this process, listing codes alongside the participants whose data related to each code; this table was iteratively refined and reorganised into the final themes reported. Data management and coding were conducted manually using Microsoft Word, without the use of dedicated qualitative data-analysis software. Disagreements among authors during coding and theme refinement were resolved through consensus, and the final thematic structure was reviewed and agreed upon by all authors prior to manuscript preparation.

Although interview analysis provided the initial thematic structure, integration across the three methodological facets proceeded iteratively rather than as three wholly separate analyses. Interview data were analysed and coded first, generating an initial thematic structure. Observational data were then examined in relation to these emerging themes, with the analysis moving iteratively between participants' reported experiences and observed practices. The observational findings largely corroborated and enriched the interview-derived themes by providing situated examples of the practices and interactional dynamics described by participants. Archival data, including institutional policy documents and patient nationality statistics, were subsequently examined in relation to the evolving thematic structure, enabling the identification of divergence between documented policies and everyday clinical practices concerning language assistance. This cross-facet process brought together individual accounts, observed practices, and institutional context to generate a cohesive set of themes informed by complementary forms of evidence. Guided by Thorne (2025) interpretive description, these thematic patterns were synthesised to move beyond mere description and generate applied, practice-relevant explanations.

This analytical strategy enabled the study to combine systematic micro-level data management with broader interpretive insights across the three methodological facets (Mason, 2011; Thorne, 2025).

## Results

The findings are organised around three main themes that show how language barriers were experienced and managed within the service. The first theme shows that language assistance relied largely on ad hoc practices despite the existence of an institutional policy, with clinicians often depending on ad hoc arrangements when communication difficulties arose. The second theme illustrates how inadequate language assistance affected clinical assessment, patient safety, emotional burden, and therapeutic engagement. The third theme presents participants’ calls for more structured, service-level responses, including institutional recognition of language barriers, professional mental health interpreters, language concordance where possible, and supplementary strategies to reduce communication breakdowns.

Table II provides an overview of the themes and subthemes identified in the analysis.

**Table II.** Themes and subthemes identified in the analysis.

Theme	Subtheme
The provision of language assistance relies on ad hoc practices despite existing institutional policies.	Clinicians rely on ad hoc and unsystematic arrangements when language barriers arise.
	Everyday practices diverge from institutional language assistance policy.
Inadequate language assistance compromises clinical assessment, patient safety, and therapeutic engagement.	Ad hoc interpreters’ limited interpreting and mental health skills compromise the accuracy of clinical assessment.
	Inconsistent access to language assistance delays care and places emotional strain on patients and clinicians.
	Interpreter-mediated encounters can disrupt trust, disclosure, and therapeutic relationships in psychiatric care.
Participants called for structured, service-level responses to language barriers.	Institutional recognition of language barriers is a necessary first step towards service reform.
	Professional mental health interpreters require linguistic, clinical, interpersonal, and ethical competencies.
	Language concordance is preferred because it enables direct, private, and emotionally safer communication.
	Supplementary strategies may reduce communication breakdowns but cannot replace professional human support.

**Theme: The provision of language assistance relies on ad hoc practices despite an existing institutional policy**

*Subtheme: Clinicians rely on ad hoc and unsystematic arrangements when language barriers arise*

Interviews and observations revealed a fundamental lack of systematic provision for language assistance within the within the Mental Health Service (MHS). In practice, psychiatrists and psychologists frequently relied on ad hoc solutions, seeking assistance from available staff members, whether clinical or non-clinical, when language barriers arose. In some cases, family members were also asked to interpret, despite concerns regarding confidentiality and accuracy. As one psychiatrist explained: “I would ask a nurse to come and help or another doctor or physician. They would come, and they would help me … sometimes even the patient’s relatives, they can, you know, become a translator and they could help” (Dr2).

This lack of systematisation was also evident in the observation data. In several sessions (Obs4, Obs6, Obs8), interpretation was provided incidentally by individuals who happened to be present, such as medical students on rotation or a physician completing a transitional placement in psychiatry. The involvement of these ad hoc interpreters was a matter of circumstance rather than planned provision. Had these individuals not been available, it remained unclear who else might have been asked to interpret.

*Subtheme: Everyday practices diverge from institutional language assistance policy*

The ad hoc approach to language assistance diverges from Hamad Medical Corporation (HMC) policy, particularly OP 4048, which outlines procedures for addressing cross-language communication needs and governs the use of the language bank. This policy defines the language bank as an approved list of HMC clinical staff fluent in languages other than English who have agreed to volunteer medical interpretation services for patients.

Despite the policy’s applicability to MHS, interviews revealed limited awareness of the language bank (Psy1, Psy2, Psy3, Dr1, Dr3). Observations further corroborated this finding, as none of the clinicians attempted to access the language bank during observed sessions. Participants identified several reasons for the limited use of the language bank. One psychologist (Psy2) noted that, unlike other departments within HMC, MHS lacks training or systems to support its use. Additionally, clinicians highlighted practical constraints, including delayed response times and the fact that language bank volunteers are not dedicated interpreters and may not be physically available when needed. Concerns were also raised about the suitability of remote interpretation for mental health encounters, particularly when discussing sensitive or highly personal issues.

**Theme: Inadequate language assistance compromises clinical assessment, patient safety, and therapeutic engagement**

*Subtheme: Ad hoc interpreters’ limited interpreting and mental health skills compromise the accuracy of clinical assessment*

Participants consistently reported that ad hoc interpreters at MHS lack professional interpreting training and mental health–specific skills, increasing the likelihood of incomplete or inaccurate interpretations (Psy1, Psy2, Dr1, Dr2, Dr3, Dr4, Dr5). Such reliance disrupts how assessments unfold, often resulting in lost clinical detail and added strain for both clinicians and patients. Several clinicians noted that professional interpreters with mental health-specific interpreting skills were rarely available, even when patients spoke languages unfamiliar to staff. As one psychiatrist stated, “we have never had any professional interpreter coming in” even in cases when a patient speaks a language no one in the hospital speaks (Dr5). Participants emphasised that inadequate interpreting skills could have serious consequences for assessment accuracy and patient safety, particularly when subtle clinical information was lost.

These concerns were most evident during risk assessment. Psychologists and psychiatrists highlighted that identifying suicidal ideation, passive death wishes, or imminent risk depends on careful attention to nuance, which untrained interpreters may fail to convey. One psychologist explained that missing such details occurred “many times” due to inadequate interpretation (Psy1), while another clinician stressed that untrained interpreters are unable to support risk assessment effectively (Dr2).

A recurring issue was the practice of summary interpreting. Clinicians who had partial understanding of Arabic described situations in which patients spoke at length, while ad hoc interpreters relayed only brief summaries. As one psychiatrist noted, “the patient is talking… for like five minutes… and then the ad hoc interpreter just summarises the thing for five seconds” (Dr5). Other clinicians and one patient reported similar experiences of minimal information transfer (Dr1, Psy1, Pt1). Observation data also reflected these accounts. In multiple sessions (Obs4, Obs5, Obs6, Obs8, Obs10), ad hoc interpreters summarised patients’ accounts, omitting clinically relevant details. In one session for example, details of stolen belongings reported by an inpatient were not conveyed to the doctor. In several cases, omissions appeared linked to side conversations between interpreters and patients.

Inaccurate interpretation also extended beyond omission. One psychiatrist described an incident in which a nurse acting as an interpreter introduced unsolicited religious commentary when interpreting for a patient with suicidal ideation, fundamentally altering the intended message (Dr2). Instead of interpreting what the doctor was saying to the patient, the “nurse goes and [says to the patient] … do you pray? … *shaitan, hatha shaitan*” (Satan this is Satan), thus adding a cultural-religious interpretation not intended by the doctor. Dr2 described the incident as being quite horrible and added that if s/he knew the language so well, such an incident could never have happened. Another clinician recalled a psychotic assessment in which a junior nurse unfamiliar with psychiatric symptoms was assigned to interpret, requiring repeated clarification and resulting in incomplete responses and a prolonged, uncomfortable interview (Dr1).

*Subtheme: Inconsistent access to language assistance delays care and places emotional strain on patients and clinicians*

It was evident across interviews and observations that inconsistent access to language assistance disrupted clinical encounters, delayed care, and increased emotional and safety-related risks for both patients and clinicians. Clinicians reported frequent difficulties securing timely support when working with Arabic-speaking patients with limited or no English proficiency. As one psychologist explained, the challenge was not only the presence of language barriers but the lack of accessible individuals to help bridge them, which affected both care delivery and clinicians’ sense of professional efficacy: “You feel as a psychologist you’re not probably doing much or you’re not being as helpful as you intend to” (Psy2). This disruption was also observed in practice. In Obs12, the absence of Arabic-speaking staff resulted in the patient being referred to another clinician at a subsequent appointment, postponing the provision of care (Obs12).

Beyond delays, participants described how interpreter unavailability directly constrained clinical work. One psychologist recalled being unable to administer the Wechsler Intelligence Scale to an Arabic-speaking inpatient due to the lack of interpretation support, resulting in reliance on a nonverbal alternative and a narrowed assessment (Psy1). Another clinician described withdrawing from the Perinatal Wellness Clinic after repeated encounters with high volumes of Arabic-speaking patients and no consistent interpreter support, noting that the situation became “difficult to manage” (Dr7). Patients described similar constraints from their perspective. One patient explained struggling to articulate her concerns in English in the absence of interpretation support, noting, “I can’t really convey my feelings to the doctor … I keep looking for the vocabulary and forget my main problem” (Pt3).

In some cases, these constraints escalated into immediate clinical risk. One psychiatrist recounted an on-call incident in the male forensic unit in which communication with an upset patient was reduced to gestures and yes/no responses because no trusted interpreter was available. De-escalation was only possible once an Arabic-speaking security staff member arrived. Reflecting on the encounter, the clinician expressed concern about not being able to provide optimal care without full linguistic access (Dr3). Similar emotional strain was described by another psychologist who waited nearly 30 minutes for interpretation support during an outpatient session, leading to feelings of helplessness and self-doubt (Psy3).

*Subtheme: Interpreter-mediated encounters can disrupt trust, disclosure, and therapeutic relationships in psychiatric care*

Participants also identified limitations inherent to the use of interpreters in psychiatric care, particularly when working with patients experiencing paranoia, delusional thinking, or heightened mistrust (Dr4, Dr5, Dr6, Dr7). In such contexts, the presence of an interpreter was described as potentially exacerbating distress and reducing patients’ willingness to speak openly. Clinicians noted that introducing a third party into encounters with already vulnerable patients could intensify feelings of insecurity. As one psychiatrist explained, patients who already “feel persecuted” may experience the presence of an interpreter as making them “feel doubly unsafe” (Dr4). This disruption of the therapeutic environment may lead patients to withhold critical information they might otherwise share in a language-concordant interaction. Another clinician described how the manner in which ad hoc interpreters communicate, including an authoritative or interrogative tone, could unintentionally escalate distress, particularly for paranoid patients (Dr5), highlighting that, in mental health care, interpretation involves not only the transfer of content but also tone, relational sensitivity, and emotional attunement.

Beyond immediate distress, clinicians emphasised the impact of interpreter use on the development of therapeutic alliance. Several participants stressed that linguistic alignment plays a key role in building trust and shared understanding. As one psychiatrist noted, “it’s always good to have a person of the same language… [to be] on the same page” (Dr4). While acknowledging that interpreters may adequately convey diagnostic or treatment-related information, clinicians described limitations in establishing sustained therapeutic relationships through mediated communication. One psychiatrist reflected that reliance on an interpreter can create a relational gap, with the interpreter becoming the primary conversational conduit rather than the clinician (Dr7). Similarly, another participant emphasised that certain clinical messages carry greater weight and therapeutic impact when delivered directly by the treating psychiatrist rather than relayed through an intermediary (Dr2).

**Theme: Participants called for structured, service-level responses to language barriers**

*Subtheme: Institutional recognition of language barriers is a necessary first step towards service reform*

Participants emphasised that addressing language barriers within the service requires an initial shift in institutional and professional awareness. They noted that the issue remains under-recognised unless it is actively discussed and acknowledged by policymakers, frontline staff, and patients. One psychologist explained, “if we don’t talk about it, people generally don’t know what’s going on” (Psy2). This recognition was viewed as a necessary precursor to the development of structured protocols, and service-level reform.

*Subtheme: Professional mental health interpreters require linguistic, clinical, interpersonal, and ethical competencies*

A dominant recommendation across interviews was the introduction of professional interpreters possessing a specific set of competencies that go beyond general language mediation.

(Dr1. Dr2. Dr3, Dr4, Dr5, Dr6, Dr7, Psy1, Psy3, Pt1, Pt2).

First, they emphasised advanced linguistic competence, noting that interpretation extends beyond basic bilingualism. Dr2 stated, “being able to speak a language is one thing and being an interpreter is another thing”. This included intralingual competence, particularly sensitivity to variation across Arabic dialects and the ability to adapt psychiatric terminology accordingly.

Second, participants stressed the importance of mental health–specific knowledge. This included familiarity with psychiatric difficulties and prior exposure to mental health settings, which were seen as essential for accurate and sensitive interpretation (Psy1, Psy2).

Third, participants emphasised that interpreters in mental health settings must possess specific interpersonal qualities that support therapeutic communication and protect patient trust. Empathy emerged as central, particularly when patients disclose distressing or deeply personal experiences. Dr7 cautioned against interpreters whose demeanour may appear dismissive or incongruent, noting that interpreters “need to be empathetic … [and] understand the person’s perspective”. Another clinician similarly stressed the importance of being “gentle, kind, [and] affectionate with the patient” (Dr5).

Beyond empathy, participants highlighted the importance of relational sensitivity and judgement, including patience, attentive listening, and the ability to establish rapport without imposing personal beliefs. Psy1 explained that effective interpretation depends on “good communication…being curious to know the patient” and engaging through open-ended rather than closed questioning.

Finally, confidentiality was consistently identified as a core ethical requirement. Given the sensitive nature of psychiatric disclosures, participants emphasised that interpreters must be trusted to respect privacy and uphold professional standards. Clinicians noted that patients often disclose information that is “not easy to share with anyone” and that trust in the interpreter’s ability to protect privacy is therefore essential (Dr2).

*Subtheme: Language concordance is preferred because it enables direct, private, and emotionally safer communication*

For many participants, the most effective form of communication in mental health care was one that eliminates the need for interpreters altogether (Dr4, Psy1, Pt2, Pt3). Clinicians noted recent efforts within MHS to better align Arabic-speaking patients with Arabic-speaking providers, while patients expressed a clear preference for direct communication without intermediaries. One patient positioned language concordance as central to privacy and emotional ease rather than as a simple communicative preference, explaining that direct interaction with the clinician felt more comfortable and protected personal boundaries (Pt3). Participants emphasised that while full concordance may not always be feasible, matching patients with clinicians who speak their language should be prioritised where possible as a core component of ethical and effective care.

*Subtheme: Supplementary strategies may reduce communication breakdowns but cannot replace professional human support*

Participants also proposed supplementary solutions to address gaps in communication. Dr2 suggested exploring AI-assisted tools to support the translation of basic psychiatric questions, particularly when miscommunication arises during consultations. However, such tools were framed as complementary rather than substitutive, with clear recognition that they cannot replace human interpreters yet. Other participants (Dr2, Dr6) proposed offering Arabic language training for non-Arabic-speaking clinicians, emphasising that even basic proficiency could enhance rapport and reduce reliance on ad hoc interpretation. Moreover, Pt1 suggested introducing a language screening tool at the point of referral or booking to assess patients’ English proficiency in advance, enabling better allocation of clinicians and interpretation resources and preventing last-minute communication breakdowns.

## Discussion

This qualitative study examined language barriers within the Mental Health Service at Hamad Medical Corporation in Qatar and shows that gaps in language assistance are not incidental but structurally embedded in everyday clinical practice. In doing so, it extends local insight while also aligning with international evidence on the clinical consequences of language discordance in mental health care. Our findings reaffirm that language discordance can undermine the quality and safety of mental health care, particularly in contexts where assessment, risk evaluation, and therapeutic work depend on nuanced communication and reliable language support (Mirza et al., 2020). This is especially significant in mental health settings, where communication is not merely a vehicle for information exchange but a core component of therapeutic engagement. As Thompson and McCabe (2012) argue, clinician-patient alliance and communication are associated with more favourable adherence, particularly where treatment depends on collaboration, agreement on the tasks of care, and discussion of treatment-related concerns. Here, the problem lies not only in the existence of language barriers themselves, but in the absence of a coordinated system that can respond to those barriers in ways that are timely, clinically appropriate, and sensitive to the demands of psychiatric care. Observational data and clinician accounts also illustrated how delays in securing interpretation support led to postponed care and, in some cases, acute clinical risk. The on-call forensic incident illustrates how effective de-escalation and risk identification depend on timely linguistic access. In other words, the timely availability of language assistance and the effective bridging of language barriers are not merely a procedural matter, but a patient safety issue as documented in broader healthcare research on language barriers (Johnstone & Kanitsaki, 2006; Van Rosse et al., 2016).

A key contribution of this study is the identification of a clear divergence between institutional policy and practice. Although HMC policy (OP 4048) formally recognises the need for structured language assistance through the language bank, most participants were either unaware of its existence or viewed it as inaccessible, impractical, or ineffective, mirroring earlier findings within HMC in Qatar, where ad hoc interpretation remains common and institutional language resources are underutilized (Abdelrahim et al., 2017; Kamal, 2015; Kanoun et al., 2024). This study extends previous work by showing how such policy–practice gaps are experienced specifically within mental health settings, where communication is central to care.

In MHS, language assistance is largely managed through ad hoc arrangements, often involving untrained clinical and non-clinical staff with limited interpreting skills. In several observed encounters, interpretation was carried out by individuals who happened to be present, including students or staff on temporary placement, indicating that interpreting was often treated as an informally assigned task rather than a protected clinical function. The reliance on ad hoc interpreters was frequently associated with incomplete or condensed translations, omission of clinically relevant detail, and side conversations that disrupted the clinician–patient interaction. These patterns are consistent with empirical evidence showing that untrained interpreters are more likely to introduce errors such as omissions, distortions, and substitutions, which may compromise clinicians’ ability to accurately assess symptoms, including disordered thought processes and delusional content (Bauer & Alegría, 2010; Mirza et al., 2020). Similar concerns have been documented in mental health settings, with empirical studies demonstrating the impact of inadequate interpretation on diagnostic accuracy (Kasraei, 2019) and clinical guidelines highlighting the associated risks for assessment and care (Miletic et al., 2006). Moreover, the use of ad hoc interpreters, particularly family members or acquaintances has been associated with reduced disclosure of sensitive psychological information, further limiting the depth and reliability of clinical assessment (Bauer & Alegría, 2010; Gartner et al., 2024).

Beyond clinical outcomes, the study foregrounds the emotional toll of unstructured language assistance on both patients and clinicians. Patients reported difficulty articulating their concerns and losing track of key issues when forced to communicate in a language in which they were not fully proficient. This aligns with qualitative evidence showing that patients in language-discordant clinical encounters often struggle to express symptoms, emotions, and concerns accurately, may become passive or disengaged during consultations, and experience uncertainty about whether critical information has been correctly conveyed or understood (Birkelund et al., 2025). Clinicians, in turn, described feelings of helplessness, professional inadequacy, and burnout when unable to provide effective care due to language constraints. Similar findings have been reported in clinical settings, where healthcare professionals experience frustration and difficulty delivering care when communication breaks down or when patients’ responses suggest misunderstanding rather than informed engagement (Patriksson et al., 2019).While previous studies have examined the emotional burden placed on interpreters themselves (Rajpoot et al., 2020), this study contributes new insight by documenting the emotional consequences of language barriers for mental health professionals and patients within a public healthcare system.

The findings also complicate any assumption that interpreter-mediated communication is, by itself, a sufficient solution in mental health contexts. Participants described situations in which the presence of an interpreter heightened mistrust and, in some cases, intensified distress, particularly among patients with paranoia. This resonates with previous research suggesting that the presence of an interpreter may, in some cases, heighten mistrust, inhibit disclosure, or be experienced as intrusive, particularly where patients fear breaches of confidentiality, third-party interference, or, in some instances, feel paranoid about the interpreter (Bauer & Alegría, 2010; Gartner et al., 2024; Hanft-Robert et al., 2023). Moreover, the findings align with existing research showing that interpreter-mediated encounters can alter therapeutic dynamics and challenge empathy, trust-building, and therapeutic alliance if roles, expectations, and communication processes are not carefully managed (Leanza et al., 2024; Pugh & Vetere, 2009; Tribe & Keefe, 2009). This may be particularly relevant in the Qatari mental health care context, where cultural expectations around family, confidentiality, gender, and disclosure may further shape how patients, clinicians, and interpreters experience the triadic encounter.

The findings suggest that language barriers within MHS cannot be addressed through individual effort alone but require systemic responses. Participants consistently emphasised the need for institutional acknowledgement of the problem, structured protocols, and investment in professional interpreters trained specifically for mental health care. Such training should encompass psychiatric terminology, risk assessment, ethical standards, sociolinguistic variation, and trauma-informed approaches. Language-concordant care where feasible should be prioritised, as both patients and clinicians expressed strong preference for direct, language-matched care. Emerging evidence suggests that language-concordant encounters are associated with improved communication, trust, and follow-up (Quigley et al., 2025). However, language concordance is not always feasible due to workforce and service constraints. This does not mean that interpreter-mediated care is inherently inferior; rather, evidence indicates that therapy delivered with professional interpreters can be effective when appropriately structured and supported (Gartner et al., 2024). Thus, while language-concordant care may represent an ideal option where available, investment in trained mental health interpreters remains essential to ensure equitable access to care.

Some additional strategies, such as language screening at referral and targeted language training for clinicians, may further strengthen service delivery by anticipating communication needs and reducing reliance on ad hoc interpreting. While AI-assisted tools may offer limited support, participants' accounts did not support relying on them independently of human interpreting and clinical judgement. Such tools were viewed as potentially useful for brief, transactional exchanges, such as simple psychiatric questions, but not as substitutes for the nuanced, relationally sensitive communication required in psychiatric assessment and therapeutic engagement. Given the interpretive and affective demands of mental health encounters, including the accurate conveyance of risk, distress, and culturally specific expressions of experience, the findings suggest AI-assisted tools should not currently be regarded as replacements for trained human interpreters, but rather as a potential supplementary aid within a broader, human-led system of language assistance. Language assistance therefore requires systemic attention and should be treated as a safety, ethical, and equity issue, in line with international standards for culturally and linguistically appropriate services (Office of Minority Health, 2021) and broader evidence showing that language barriers contribute to disparities and inequities in healthcare (Diamond et al., 2019). Our findings contribute mental health–specific evidence from Qatar, showing that language barriers are central to the delivery of care rather than peripheral concerns. In order to address these barriers, there is a need for coordinated institutional reform that moves beyond incidental solutions toward clinically appropriate language assistance within public mental health services.

## Limitations and future avenues for research

This study has limitations. The relatively small sample size limits the breadth of perspectives captured, and future studies would benefit from including larger and more diverse participant groups, particularly patients and interpreters, to strengthen analytical depth. The imbalance between the clinician and patient samples may have weighted the findings towards a more clinician-centred account. With only three patient participants, the findings may not capture the full diversity of patients’ experiences of language barriers in mental healthcare. As the study was conducted exclusively within MHS, the findings may not be fully transferable to other clinical settings or departments within HMC. The qualitative themes identified here could be extended through complementary quantitative approaches, such as surveys or structured questionnaires, to examine their prevalence and associations. Future research may also evaluate the outcomes of language-concordant care and pilot interventions, including AI-assisted interpreting tools, Arabic language and culture–focused training for clinicians, and language screening mechanisms to support more appropriate allocation of clinicians, patients, and interpreters. Future studies should also extend this line of enquiry to other linguistically diverse patient populations, including Asian and African language groups.

Current initiatives to professionalise interpreting in Qatar, such as the *Bridging the Gap* medical interpreter training licensed by the Cross-Cultural Health Care Programme and delivered by Weill Cornell Medicine in Qatar (Weill Cornell Medicine-Qatar, 2025), represent important progress toward culturally and emotionally responsive interpreting services. Expanding similar programmes and embedding their principles within institutional policy frameworks may be critical to sustained improvement.

## Conclusion

Our findings highlight the need for systemic reform in the provision of language assistance within the Mental Health Service at Hamad Medical Corporation. They show that the prevailing reliance on ad hoc or incidental interpreters can undermine safety, clinical accuracy, and therapeutic rapport while placing emotional and professional strain on patients and clinicians. Institutional acknowledgement of the problem, investment in mental health-specific interpreter training and the prioritisation of language-concordant care wherever possible are needed to address these deficiencies. The development of a structured and culturally competent interpreting service, supported by clear policies, consistent interpreter allocation, and clinician training, is essential to improving psychiatric care and strengthening trust, engagement, and treatment outcomes for linguistically diverse patients.

## Data Availability

The qualitative data generated and analysed during this study are not publicly available due to ethical restrictions and the need to protect participant confidentiality. The data consist of interview, observational materials, and institutional data from a mental health care setting, which may contain sensitive and potentially identifiable information. Requests for access to de-identified data may be directed to the corresponding author at DAlkhatib@hamad.qa, subject to institutional approval and compliance with relevant ethical and confidentiality requirements.

## Endnote

^1^
Mason (2011) describes facet methodology through the metaphor of a cut gemstone, where different facets cast and refract light on the overall object of enquiry. In this study, interviews, observations, and archival materials were treated as distinct but related facets that illuminated different dimensions of language barriers in mental health care.
